# Association of lower urinary tract syndrome with peripheral arterial occlusive disease

**DOI:** 10.1371/journal.pone.0170288

**Published:** 2017-03-16

**Authors:** Wei-Yu Lin, Karl-Erik Andersson, Cheng-Li Lin, Chia-Hung Kao, Hsi-Chin Wu

**Affiliations:** 1 Division of Urology, Department of Surgery, Chang Gung Memorial Hospital, Chia-Yi, Taiwan; 2 Department of Medicine, Chang Gung University, Taoyuan, Taiwan; 3 Chang Gung University of Science and Technology, Chia-Yi, Taiwan; 4 Department of Medicine and Graduate Institute of Clinical Medical Sciences, College of Medicine, Chang Gung University, Taiwan; 5 Wake Forest Institute for Regenerative Medicine, Wake Forest University School of Medicine, Winston-Salem, North Carolina, United States of America; 6 Management Office for Health Data, China Medical University Hospital, Taichung, Taiwan; 7 College of Medicine, China Medical University, Taichung, Taiwan; 8 Graduate Institute of Clinical Medical Science and School of Medicine, College of Medicine, China Medical University, Taichung, Taiwan; 9 Department of Nuclear Medicine and PET Center, China Medical University Hospital, Taichung, Taiwan; 10 Department of Bioinformatics and Medical Engineering, Asia University, Taichung, Taiwan; 11 Department of Urology, China Medical University Beigang Hospital, Taichung, Taiwan; University of Catania, ITALY

## Abstract

**Purpose:**

To describe atherosclerosis may lead to chronic bladder ischemia, eventually resulting in lower urinary tract syndrome (LUTS), and peripheral arterial occlusive disease (PAOD). We investigated the association of LUTS with PAOD.

**Methods:**

This nationwide population-based cohort study was based on data from the Taiwan National Health Insurance Database from 2000 to 2010; follow-up lasted until the end of 2011. We identified patients with newly diagnosed LUTS by using International Classification of Diseases, Ninth Revision, Clinical Modification codes.

**Results:**

In total, 36,042 and 36,042 patients were enrolled in LUTS and non-LUTS cohorts, respectively. After adjustment for age, sex, and comorbidities, the risk of subsequent PAOD was 1.36-fold higher [95% confidence interval (CI) = 1.26–1.46] in the LUTS cohort than in the non-LUTS cohort. The adjusted risk of PAOD was the highest in patients with LUTS without any comorbidity [adjusted hazard ratio (aHR) = 1.93, 95% CI = 1.54–2.41]. The age-specific relative risk of PAOD was significantly higher in all age groups, particularly in those aged <49 years (aHR = 1.80, 95% CI = 1.39–2.34], in the LUTS cohort than in the non-LUTS cohort.

**Conclusion:**

LUTS is a risk factor for PAOD. Physicians should consider the possibility of underlying PAOD in patients with LUTS aged <49 years and without cardiovascular comorbidities. Additional studies developing strategies for decreasing the risk of PAOD are warranted.

## Introduction

In 2008, approximately 45.2% of the worldwide population (4.3 billion) was affected by at least one lower urinary tract syndrome (LUTS) [1]. Patients with LUTS seeking medical help increased annually from 2000 to 2009 in Taiwan [2].

Atherosclerosis can lead to chronic bladder ischemia, which may be crucial in the development of LUTS [3–7]. In addition, atherosclerosis can lead to peripheral arterial occlusive disease (PAOD), which affects >202 million people worldwide [7–8]. However, up to 50% of patients with PAOD are asymptomatic [9–10]. Because LUTS and PAOD share certain risk factors, such as metabolic syndromes (hypertension, hyperlipidemia, and diabetes), obesity, smoking, and advanced age, we hypothesized that LUTS is a sentinel symptom in patients with PAOD [6, 10–15].

Diseases caused by atherosclerosis are the leading cause of illness and death for both men and women. Hence, we would like to illustrate our hypothesis in both genders. We performed a nationwide population-based cohort study in Taiwan to investigate whether LUTS precedes PAOD.

## Methods

### Data source

This retrospective cohort study was based on data obtained from the Longitudinal Health Insurance Database 2000 (LHID2000). The LHID2000, made available for research, contains 1 million beneficiaries from the National Health Insurance (NHI) program. The NHI program was implemented in 1995 in Taiwan and has been providing health insurance to > 99% (23 million) of the population (http://nhird.nhri.org.tw/en/index.html). The disease diagnoses are created on the International Classification of Diseases, Ninth Revision, Clinical Modification (ICD-9-CM) codes.

### Data availability statement

The dataset used in this study is held by the Taiwan Ministry of Health and Welfare (MOHW). The Ministry of Health and Welfare must approved our application to access this data. Any researcher interested in accessing this dataset can submit an application form to the Ministry of Health and Welfare requesting access. Please contact the staff of MOHW (Email: stcarolwu@mohw.gov.tw) for further assistance. Taiwan Ministry of Health and Welfare Address: No.488, Sec. 6, Zhongxiao E. Rd., Nangang Dist., Taipei City 115, Taiwan (R.O.C.). Phone: +886-2-8590-6848. All relevant data are within the paper.

### Ethics statement

The NHIRD encrypts patient personal information to protect privacy and provides researchers with anonymous identification numbers associated with relevant claims information, including sex, date of birth, medical services received, and prescriptions. Therefore, patient consent is not required to access the NHIRD. This study was approved to fulfill the condition for exemption by the Institutional Review Board (IRB) of China Medical University (CMUH104-REC2-115-CR1). The IRB also specifically waived the consent requirement.

### Sampled patients

We included patients with newly diagnosed LUTS [including (A) voiding symptoms, such as retention of urine (ICD-9-CM code 788.2), splitting and slowing of urine stream (ICD-9-CM code 788.6), and postvoid dribbling (ICD-9-CM code 788.35); (B) storage symptoms, such as frequency and polyuria (ICD-9-CM code 788.4), stress urinary incontinence in women (ICD-9-CM code 625.6) and men (ICD-9-CM code 788.32), urgency incontinence (ICD-9-CM code 788.31), bladder hypertonicity (ICD-9-CM code 596.51), nocturnal enuresis (ICD-9-CM code 788.36), nocturia (ICD-9-CM code 788.43), and mixed incontinence (ICD-9-CM code 788.33); and (C) benign prostate hyperplasia (BPH) in men (ICD-9-CM code 600)] between January 1, 2000, and December 31, 2010, into the LUTS cohort. The date of first LUTS diagnosis was defined as the index date. Patients who had a history of PAOD (ICD-9-CM codes 440.2, 440.3, 440.8, 440.9, 443, 444.22, 444.8, 447.8, and 447.9) before the index date or who were aged < 20 years were excluded. For each patient with LUTS, we included a patient without LUTS frequency matched by age (every 5-year span); sex; index year; and baseline comorbidities, namely diabetes (ICD-9-CM code 250), hypertension (ICD-9-CM codes 401–405), hyperlipidemia (ICD-9-CM code 272), chronic obstructive pulmonary disease (COPD; ICD-9-CM codes 491, 492, and 496), heart failure (ICD-9-CM code 428), coronary artery disease (CAD; ICD-9-CM codes 410–414), stroke (ICD-9-CM codes 430–438), and asthma (ICD-9-CM code 493) into the non-LUTS cohort. Finally, 36,042 and 36,042 patients were included in LUTS and non-LUTS cohorts, respectively. The study patients were followed until PAOD diagnosis, withdrawal from the NHI program, death, or end of 2011, whichever occurred earlier.

### Statistical analysis

The distribution of sex, age, and comorbidity was compared between both cohorts. The difference between 2 cohorts were tested by Chi-square test for categorical variables and two sample t-test for continuous variables. The cumulative incidence of PAOD in both cohorts was estimated using Kaplan–Meier curves and the difference in curves were compared using the log-rank test. The incidence density rate of PAOD was evaluated in both cohorts (per 1000 person-years). The risk of PAOD in the LUTS cohort relative to that in the non-LUTS cohort after stratification by age, sex group, and comorbidity was evaluated through unavailable and multivariable Cox proportional hazard regression models. Multivariable Cox models were mutually adjusted for age and comorbidities, namely diabetes, hypertension, hyperlipidemia, COPD, heart failure, CAD, stroke, and asthma. All analyses were performed using the Statistical Analysis Software Version 9.4 (SAS Institute Inc., Carey, NC). The significance level was set at 0.05 for the two-tailed tests.

## Results

**Fig 1:**
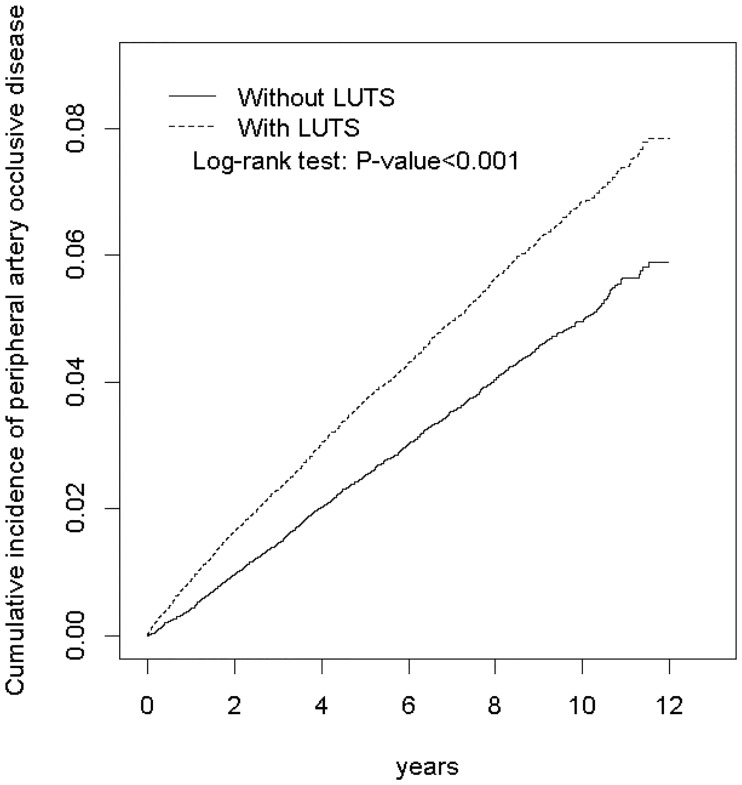
Cumulative incidence of peripheral artery occlusive disease compared between with and without LUTS cohorts using the Kaplan-Meier method.

The distribution of sex, age, and comorbidities in both cohorts is presented in Table 1. Among 36042 patients with LUTS, over 98.7% of patients with LUTS (n = 35584) were on treatment. No statistical difference was observed in the distribution of sex, age, and comorbidities between the cohorts, except for heart failure and asthma. The mean age of patients in LUTS and non-LUTS cohorts was 59.9 ± 15.0 and 59.4 ± 14.8 years, respectively. In both cohorts, most patients were men (71.1%) and aged >65 years (41.6%). Furthermore, in both cohorts, the major comorbidity was hypertension (47.7%), followed by hyperlipidemia (25.3%), stroke (23.7%), and CAD (23.0%). The average follow-up period (years) in LUTS and non-LUTS cohorts was 6.55 and 6.47 years, respectively (data not shown). The cumulative incidence of PAOD was higher in the LUTS cohort than in the non-LUTS cohort (Fig 1).

**Table 1:** Demographic characteristics and comorbidity in patient with and without LUTS.

	LUTS		
	No	Yes	
Variable	N = 36042	N = 36042	p-value
**Sex**	N (%)	N (%)	0.01
Female	10712(29.7)	10409(28.9)	
Male	25330(70.3)	25633(71.1)	
**Age, mean(SD)**	59.4(14.8)	59.9(15.0)	0.001
**Stratify age**			0.12
≤49	9039(25.1)	9126(25.3)	
50–65	12193(33.8)	11932(33.1)	
65+	14810(41.1)	14984(41.6)	
**Comorbidity**			
Diabetes	4726(13.1)	4837(13.4)	0.22
Hypertension	16954(47.0)	17192(47.7)	0.08
Hyperlipidemia	8930(24.8)	9099(25.3)	0.15
COPD	6337(17.6)	6151(17.1)	0.07
Heart failure	1247(3.46)	1046(2.90)	0.001
CAD	8216(22.8)	8277(23.0)	0.59
Stroke	8416(23.4)	8538(23.7)	0.28
Asthma	2974(8.25)	2820(7.82)	0.03

Chi-Square Test; #: Two sample T-test

The overall incidence density rate of PAOD in LUTS cohort was 7.26 per 1000 person-years and in non-LUTS cohort 5.14 per 1000 person-years, respectively. After adjustment for age and comorbidities of hypertension, diabetes, COPD, hyperlipidemia, heart failure, stroke, CAD, and asthma, the risk of PAOD was higher in the LUTS cohort than in the non-LUTS cohort [adjusted hazard ratio (aHR) = 1.36, 95% confidence interval (CI) = 1.26–1.46; Table 2]. The sex-specific relative risk of PAOD was significantly higher in both women (aHR = 1.37, 95% CI = 1.18–1.58) and men (aHR = 1.08, 95% CI = 1.01–1.16) in patients with LUTS than patients without LUTS. The age-specific relative risk of PAOD was significantly higher in all age groups in patients with LUTS than patients without LUTS. The relative risk of PAOD was higher in the LUTS cohort than in the non-LUTS cohort for both patients without comorbidity (aHR = 1.93, 95% CI = 1.54–2.41) and with comorbidity (aHR = 1.32, 95% CI = 1.22–1.43).

**Table 2:** Comparison of incidence and hazard ratio of peripheral artery occlusive disease stratified by sex, age and comorbidity between with and without LUTS patients.

	Without LUTS					With LUTS				
Variable	Event	PY	Rate^#^	Crude HR* (95% CI)	Adjusted HR^†^ (95% CI)	Event	PY	Rate^#^	Crude HR* (95% CI)	Adjusted HR^†^ (95% CI)
**All**	1198	233180	5.14	1(Reference)	1(Reference)	1714	236146	7.26	1.41(1.31, 1.52)***	1.36(1.26, 1.46)***
**Sex**										
Female	331	72138	4.59	1(Reference)	1(Reference)	407	70069	5.81	1.27(1.10, 1.47)**	1.37(1.18, 1.58)***
Male	867	161042	5.38	1(Reference)	1(Reference)	1307	166077	7.87	1.46(1.34, 1.59)***	1.35(1.24, 1.47)***
**Stratify age**										
≤49	88	64523	1.36	1(Reference)	1(Reference)	160	65400	2.45	1.79(1.38, 2.32)***	1.80(1.39, 2.34)***
50–65	411	83294	4.93	1(Reference)	1(Reference)	526	82243	6.40	1.30(1.14, 1.47)***	1.31(1.15, 1.49)***
65+	699	85363	8.19	1(Reference)	1(Reference)	1028	88503	11.6	1.42(1.29, 1.56)***	1.39(1.26, 1.53)***
**Comorbidity**^**‡**^										
No	114	81751	1.39	1(Reference)	1(Reference)	225	82594	2.72	1.95(1.56, 2.45)***	1.93(1.54, 2.41)***
Yes	1084	151429	7.16	1(Reference)	1(Reference)	1489	153553	9.70	1.36(1.25, 1.47)***	1.32(1.22, 1.43)***

Rate^#^, incidence rate, per 1,000 person-years; Crude HR*, crude hazard ratio

Adjusted HR^†^: multivariable analysis including age, and comorbidities of diabetes, hypertension, hyperlipidemia, COPD, heart failure, CAD, stroke, and asthma

**p<0.01,

***p<0.001

Comorbidity^‡^: Patients with any one of the comorbidities diabetes, hypertension, hyperlipidemia, obesity, COPD, heart failure, CAD, stroke, and asthma were classified as the comorbidity group

The risk factors for PAOD analyzed using Cox models are presented in Table 3. The aHR of PAOD increased by 1.03-fold with age (every year; 95% CI = 1.03–1.04). In multivariable model, the risk of Parkinson’s disease was 7% higher in men than in women (aHR = 1.07, 95% CI = 1.03–1.12) and was higher in patients with comorbidities, namely diabetes (aHR = 1.76, 95% CI = 1.61–1.92), hypertension (aHR = 1.51, 95% CI = 1.38–1.65), hyperlipidemia (aHR = 1.20, 95% CI = 1.11–1.30), COPD (aHR = 1.16, 95% CI = 1.06–1.27), CAD (aHR = 1.27, 95% CI = 1.17–1.38), and stroke (HR = 1.25, 95% CI = 1.16–1.36).

**Table 3:** HR of peripheral artery occlusive disease in association with sex, age, and comorbidities in univariable and multivariable Cox regression models.

	Crude*		Adjusted^†^	
Variable	HR	(95% CI)	HR	(95% CI)
LUTS	1.41	(1.31, 1.52)***	1.36	(1.26, 1.46)***
Sex (Women vs Men)	1.28	(1.18, 1.39)***	1.04	(0.96, 1.13)
Age, years	1.04	(1.04, 1.05)***	1.03	(1.03, 1.04)***
**Baseline comorbidities (yes vs no)**				
Diabetes	2.51	(2.31, 2.73)***	1.76	(1.61,1.92)***
Hypertension	2.84	(2.62, 3.07)***	1.51	(1.38, 1.65)***
Hyperlipidemia	1.74	(1.61, 1.87)***	1.20	(1.11, 1.30)***
COPD	1.71	(1.57, 1.86)***	1.16	(1.06, 1.27)**
Heart failure	2.15	(1.82, 2.54)***	1.12	(0.94, 1.33)
CAD	2.24	(2.08, 2.41)***	1.27	(1.17, 1.38)***
Stroke	2.06	(1.91, 2.22)***	1.25	(1.16, 1.36)***
Asthma	1.35	(1.19, 1.52)***	0.99	(0.87, 1.13)

Crude*, relative hazard ratio;

Adjusted^†^: multivariable analysis including age, and comorbidities of diabetes, hypertension, hyperlipidemia, COPD, heart failure, CAD, stroke, and asthma;

**p<0.01,

***p<0.001

## Discussion

PAOD is one of the most fatal diseases; however, it is often ignored [9, 16]. Even without a history of ischemic stroke or myocardial infarction, patients with PAOD have the same risk of death as do patients with related cardiovascular disease (CVD) [10, 17–19]. LUTS is considered the initial manifestation of underlying PAOD. Diabetes could be as crucial link between LUTS and PAOD because the close association between LUTS vs. diabetes and diabetes vs. PAOD have been recognized [20, 21]. Identification of a predictive symptom can allow early intervention and thus decrease complications resulting from the disease [22]. This can be extremely crucial for patients without regular and adequate medical assessments of CVD risk factors [23].

LUTS comprise a variety of etiologies from benign disease to malignance. In 2008, approximately 45.2% of the worldwide population (4.3 billion) was LUTS. Patients with LUTS seeking medical help increased annually from 2000 to 2009 in Taiwan. Therefore, it is important to investigate if LUTS could be regarded as a sentinel symptom in patients with critical health issues, which are often ignored such as PAOD Although LUTS and CAD share the same risk factors, including obesity, tobacco use, physical inactivity, diabetes, hypertension, and hyperlipidemia, the association of LUTS with CAD remains controversial [23–27]. By contrast, the relationship between LUTS and PAOD has been reported in animal studies and cohort observations; however, this relationship has not been reported in a nationwide population-based cohort study.

In clinical studies, Pinggera and colleagues reported that elderly patients with LUTS had a significant decrease in bladder blood flow compared with that in asymptomatic young individuals [1, 28]. In addition, they reported that α1-adrenergic receptor (AR) antagonists improved symptoms in patients with LUTS and bladder blood flow [29], suggesting that α1-AR antagonist drugs might ameliorate LUTS by increasing the bladder blood flow.

In animal models, atherosclerosis induced pelvic ischemia and caused functional and structural alteration of the bladder muscle [6, 30–32]. Anatomically, the vascular supply to the lower urinary tract is primarily from the iliac arteries. Therefore, atherosclerotic obstructive changes distal to the aortic bifurcation can affect the distal vasculature and lower urinary tract blood flow [1, 7]. Bladder ischemia can precede PAOD because of the easy obstruction caused by atherosclerosis in most distal arterial branches.

The NHIRD provides opportunities to retrospectively investigate the association of LUTS with subsequent PAOD. In both cohorts, most patients were men (71.1%) and aged >65 years (41.6%); this is because patients diagnosed with BPH were also included. However, risk of PAOD was significantly higher in both women (aHR = 1.37, 95% CI = 1.18–1.58) and men (aHR = 1.08, 95% CI = 1.01–1.16) with LUTS.

In the data of NHIRD, there are no further specific information about the pelvic arterial situation. However, the majority of the PAOD occurred in lower limbs with a much greater numbers than the upper limbs. Therefore, to some degree, the diagnosis of PAOD properly reflect the poor pelvic artery situation.

After adjustment for age and comorbidities, namely diabetes, hypertension, hyperlipidemia, COPD, heart failure, CAD, stroke, and asthma, the risk of PAOD was higher in the LUTS cohort than in the non-LUTS cohort (aHR = 1.36, 95% CI = 1.26–1.46). Moreover, the relative risk of PAOD was higher in the LUTS cohort than in the non-LUTS cohort for both patients without comorbidity (aHR = 1.93, 95% CI = 1.54–2.41) and with comorbidity (aHR = 1.32, 95% CI = 1.22–1.43). Furthermore, the risk of PAOD was higher in patients aged <49 years in the LUTS cohort (aHR = 1.80, 95% CI = 1.39–2.34]. Therefore, clinicians should consider the possibility of underlying PAOD in patients with LUTS aged <49 years and without any cardiovascular comorbidity.

Our study has several limitations that should be addressed. First, the diagnosis codes (i.e., the ICD-9-CM codes) may be incorrect, and information on the accuracy of the codes for LUTS is lacking. LUTS is perhaps undercoded because it is a symptom diagnosis. Thus, this error might underestimate the effect of LUTS on subsequent PAOD [24].

Second, the diagnoses of LUTS, PAOD, and other comorbidities were based on ICD-9-CM codes and thus misclassification is possible. However, the use of ICD-CM-9 codes for diagnosing chronic diseases has been validated in previous national cohort studies [10, 24, 33–35]. The NHIRD covers a highly representative sample of Taiwan’s general population because the reimbursement policy is universal and operated by a single-buyer, the government in Taiwan. Moreover, the National Health Insurance Bureau of Taiwan reviews charts, confirms medical charges, and executes heavy penalties for malpractice and inappropriate charges. The definition of PAD was based on ICD- 9-CM codes determined by physicians after strict assessments in the reimbursement process based on pathological, imaging, and laboratory data. These checks and balances are expected to ensure accurate coding.

Third, the NHIRD lacks information on some critical cardiovascular risk factors such as smoking, obesity, BMI, alcoholism, exercise, and dietary habits. We have included hypertension, diabetes, and hyperlipidemia to adjust for the influence of BMI and obesity. To minimize the potential confounding effect of smoking, we adjusted for smoking-related diseases such as COPD, asthma, and stroke; these diseases were used in previous studies [36, 37]. However, with these limitations, a high prevalence of LUTS as a sentinel symptom in patients with occult PAOD should be considered.

The suggestions of this study are important. With the accessibility of pharmacotherapy in effect, a growing number of people are pursuing care for LUTS. In 2008, approximately 45.2% of the worldwide population (4.3 billion) was affected by at least one LUTS, with an age-related increase in both sexes [1, 38]. Our data suggest that patients with LUTS have an approximately 1.36-fold higher risk of subsequent PAOD than do patients without LUTS. With up to 50% of patients with PAOD being asymptomatic, the risk of death in patients with PAOD is the same as that in patients with CVD. This analysis advises that the initial appearance of patients with LUTS, particularly of those aged <49 years old or without any comorbidity, should rapid the assessing physician to screen for standard PAOD risk factors, and intervention for PAOD should be accordingly initiated.

## Conclusion

This nationwide population-based cohort study indicated that LUTS is associated with subsequent PAOD, particularly in patients aged <49 years and without cardiovascular comorbidities. Physicians should consider the possibility of PAOD in patients with LUTS. Additional studies on disease screening and early intervention are warranted to prevent the subsequent complications of PAOD in patients with LUTS.

## Abbreviations

aHR: adjusted hazard ratio
CI: confidence interval
ICD-9-CM: International Classification of Diseases, Ninth Revision, Clinical Modification
LHID2000: Longitudinal Health Insurance Database 2000
LUTS: lower urinary tract syndrome
PAOD: peripheral arterial occlusive disease
